# Advances in fetal and neonatal neuroimaging and everyday exposures

**DOI:** 10.1038/s41390-024-03294-1

**Published:** 2024-06-14

**Authors:** Alexandra Lautarescu, Alexandra F. Bonthrone, Brendan Bos, Ben Barratt, Serena J. Counsell

**Affiliations:** 1 Department of Perinatal Imaging and Health, Centre for the Developing Brain, School of Biomedical Engineering and Imaging Sciences, King’s College London, London, UK; 2 Department of Psychology, Institute of Psychiatry, Psychology and Neuroscience, King’s College London, London, UK; 3 MRC Centre for Environment and Health, Imperial College London, London, UK

## Abstract

**Abstract:**

The complex, tightly regulated process of prenatal brain development may be adversely affected by “everyday exposures” such as stress and environmental pollutants. Researchers are only just beginning to understand the neural sequelae of such exposures, with advances in fetal and neonatal neuroimaging elucidating structural, microstructural, and functional correlates in the developing brain. This narrative review discusses the wide-ranging literature investigating the influence of parental stress on fetal and neonatal brain development as well as emerging literature assessing the impact of exposure to environmental toxicants such as lead and air pollution. These ‘everyday exposures’ can co-occur with other stressors such as social and financial deprivation, and therefore we include a brief discussion of neuroimaging studies assessing the effect of social disadvantage. Increased exposure to prenatal stressors is associated with alterations in the brain structure, microstructure and function, with some evidence these associations are moderated by factors such as infant sex. However, most studies examine only single exposures and the literature on the relationship between in utero exposure to pollutants and fetal or neonatal brain development is sparse. Large cohort studies are required that include evaluation of multiple co-occurring exposures in order to fully characterize their impact on early brain development.

**Impact:**

- Increased prenatal exposure to parental stress and is associated with altered functional, macro and microstructural fetal and neonatal brain development.
- Exposure to air pollution and lead may also alter brain development in the fetal and neonatal period.
- Further research is needed to investigate the effect of multiple co-occurring exposures, including stress, environmental toxicants, and socioeconomic deprivation on early brain development.

## Introduction

The development of the human central nervous system is a complex process which includes orchestrated events such as neural proliferation, differentiation, migration, and cortical organization and is influenced by both genotype and early environment.^1^ In line with Barker’s “Developmental Origins of Health and Disease” hypothesis”,^2^ stimuli or insults during critical periods of in utero development have the potential to affect brain morphology and function and lead to adverse outcomes later in life.

Recent advances in neuroimaging have enabled in vivo investigations of the fetal and neonatal brain, offering novel insights into the relationships between prenatal exposures and early brain development in the absence of postnatal influences. Exposures with potential to impact early brain development are wide-ranging and often co-occurring. In this narrative review, we discuss how prenatal exposure to parental mental ill-health and environmental toxicants are related to brain structure and function. The focus of this narrative review was determined based on the high prevalence of these ‘everyday exposures’. Maternal depression, anxiety, and stress impact more than 1 in 5 pregnancies,^3^ with prevalence rates as high as 64% during the COVID-19 pandemic.^4^ Environmental toxicants are also a common exposure, with UNICEF estimating that over 100 million infants and 300 million children are exposed to pollution levels that exceed WHO recommended limits^5^ and around 95% of the world’s population lives in regions with unhealthy air pollution levels.^6^ As socioeconomic adversity is frequently associated with these exposures,^7^ we also include a brief discussion on the relationship between socioeconomic deprivation and early brain development. We note that gendered terms such as “mothers” and “fathers” are being used in this review to reflect the composition of the included samples. In addition, the neurobiological changes (beyond those revealed through MRI) associated with prenatal stress exposure (e.g., HPA axis, inflammation, the neurobiological pathway of vulnerability) have been discussed in other reviews^8–10^ and are therefore not addressed here.

## MRI approaches to assess early brain development

The following section discusses advances in fetal and neonatal magnetic resonance imaging (MRI) and analyses which have been used to characterize the relationship between everyday exposures and brain development.

### Advances in fetal and neonatal MRI

MRI is increasingly used to non-invasively acquire detailed images of the developing brain in utero and in the early postnatal period. Advanced MRI approaches can provide quantitative assessments of tissue morphology, microstructure, and functional activity. Importantly, fetal and neonatal MRI allows researchers to characterize the effects of prenatal exposures on brain development while minimizing the impact of postnatal exposures. Acquisition protocols have been developed specifically for neonates using neonatal head coils, multiband acceleration methods and motion tolerant sequence designs.^11^ In addition, specialized post-processing techniques designed to remove motion artefacts and model the unique properties of the developing brain have also emerged (see Dubois and colleagues^12^ for a review of neonatal MRI). Fetal brain MRI poses additional unique challenges including motion from mother and fetus, artefacts from maternal bowel and pelvic organs, transient fetal brain structures, changing tissue properties, and ensuring safety and maternal well-being. Methodological advances in fetal MR which aim to address some of these challenges are reviewed elsewhere.^13–15^ However, large-scale fetal MRI studies are increasing, including the recently completed developing human connectome project (dHCP) (http://www.developingconnectome.org/). These datasets are crucial to characterize the effect of everyday exposures on early brain development across gestation.

### Structural MRI

**Fig. 1:**
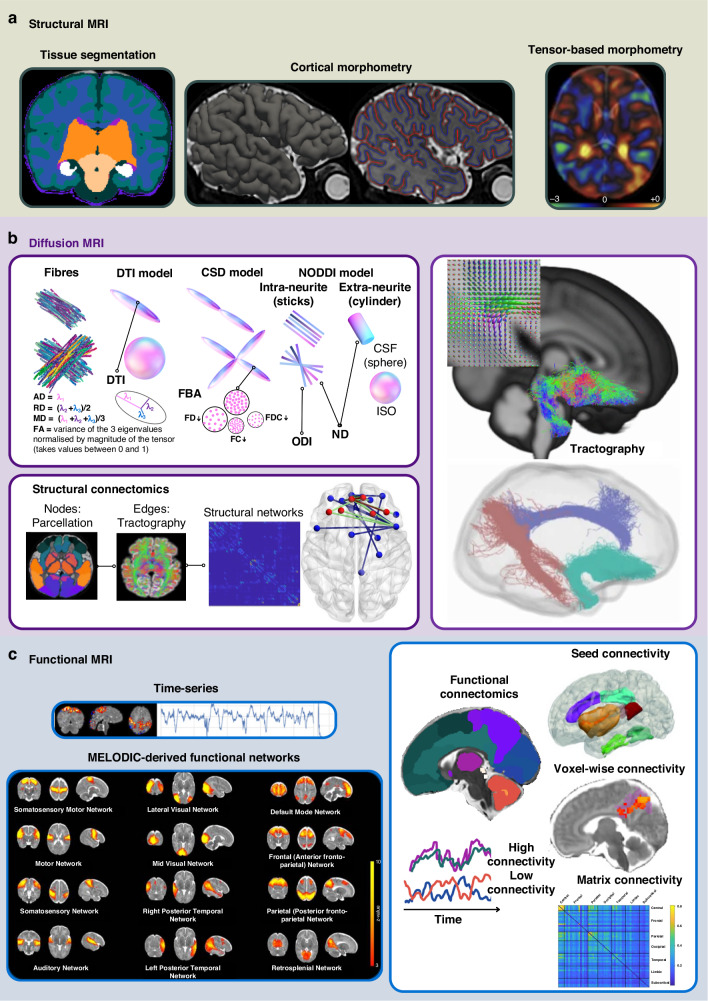
Advanced neuroimaging approaches. **a** Structural MRI: Left and Center, Brain segmentation and cortical morphometry of a neonate from dHCP data release 3^44^; Right, Tensor-based morphometry t-statistic map adapted from Lautarescu and colleagues.^57^ **b** Diffusion MRI: top left, diagram of DTI, CSD, and NODDI models; Bottom left, structural connectomics network construction and edge-wise analysis, adapted from Bonthrone and colleagues^152^; Right, tractography reconstruction of the uncinate fasciculus with an illustration of fiber orientation distributions in a region of crossing fibers and glass brain illustration of uncinate fasciculus, dorsal and ventral cingulum tractography reconstructions, adapted from Lautarescu and colleagues.^71^ **c** Functional MRI: Top left, Illustration of time-series from the sensorimotor network adapted from Fitzgibbon and colleagues^153^; bottom left, MELODIC-derived resting-state functional networks adapted from Ciarrusta and colleagues^154^; right, Functional connectivity analysis including a neonatal adaptation of the AAL atlas,^155^ and seed connectivity and voxel-wise connectivity adapted from Ciarrusta and colleagues^156^; figure based on Kwon and colleagues.^157^ All images adapted from papers published under Creative Commons Attribution Licence CC BY 4.0 (https://creativecommons.org/licenses/by/4.0/).

High-resolution 3D structural brain MRI data, based on T_1_ or T_2_ weighting, can be used to investigate associations between prenatal everyday exposures and total or regional brain size and shape. Total and regional brain volumes can be calculated from neonatal and fetal specific brain tissue classification methods (based on image signal intensity) which automatically delineate different brain regions^16^ (Fig. 1a, left). Voxel-wise shape and volume changes can be assessed with deformation methods such as tensor-based morphometry (Fig. 1a, center), which localizes structural variation more precisely than segmentation methods and accounts for global scaling effects (i.e., overall head and brain size).

The developing cortex can be assessed using neonatal and fetal specific cortical surface reconstruction pipelines such as the dHCP surface pipeline^17^ or iBEAT v2.0^18^ (Fig. 1a, right). Using reconstructed surfaces, the morphometric properties of the cortex can be characterized at a global, regional and vertex-wise level. Measures capturing the shape and size of the cortex include cortical thickness, total surface area, gyrification index, curvature, and sulcal depth.

Associations between everyday exposures before birth and measures of volume, shape and cortical morphometry can be characterized across the whole brain or within specific structures chosen a-priori. For example, in the prenatal stress literature limbic structures such as the amygdala and hippocampus are commonly assessed.

### Diffusion MRI

Diffusion MRI (dMRI) enables white and gray matter microstructure and fiber-specific morphology to be probed (for review see Pecheva, Kelly and colleagues^19^). dMRI measures the displacement of water molecules in tissue over time. In cerebrospinal fluid (CSF), displacement is equal in all directions (isotropic diffusion). However, in tissue with an ordered structure such as white matter, water diffusion is impeded by axonal membranes, and is directionally dependent (anisotropic) on the orientation of the underlying fibers.^20–22^ Mathematical models are used to infer quantitative information about underlying tissue from the measured dMRI signal (See Fig. 1b for details of dMRI modeling).

The most commonly used model in studies of early life exposures is diffusion tensor imaging (DTI), which is fitted voxel-wise across the brain. It provides scalar rotationally invariant metrics,^23^ derived from three eigenvalues (λ_1_, λ_2_, λ_3_) which are, by definition, independent of orientation. Metrics characterizing the diffusion properties can be calculated from the tensor, including fractional anisotropy, and axial, radial and mean diffusivity (see Fig. 1b top left for details).^24^ While DTI has facilitated many insights into the relationship between everyday exposures and the developing brain, a major limitation is that DTI does not accurately represent the underlying microstructure in the presence of multiple fiber populations with different orientations (‘crossing fibers’). DTI metrics also lack tissue specificity, as these measures can be affected by both intracellular and extracellular water diffusion and non-neuronal structures.

High angular resolution diffusion imaging (HARDI) acquisitions with high b-values and acquisitions using multiple b-values (termed multi-shell) are required for more advanced analyses that characterize the underlying tissue with greater specificity. These approaches have long acquisition times which have limited their use in fetuses and unsedated neonates. However, advances in MRI acquisition techniques have increased the use of HARDI and multiple b-value acquisitions in this difficult to image population.^25^

Constrained spherical deconvolution (CSD)^26^ is a dMRI model designed to overcome the limitations of DTI by modeling several diffusion orientations within a voxel (Fig. 1b). These diffusion orientations can be isolated as ‘fixels’ (fiber orientations within a voxel).^27^ Fixel-based metrics of fiber-specific microstructure (apparent fiber density, FD) and morphology (fiber cross-section, FC) can be extracted. Fiber density modulated by cross section (FDC), calculated as the product of FD and FC, serves as a proxy of a fiber bundle’s capacity to relay information.^27^

Other dMRI models, such as the neurite orientation dispersion and density imaging (NODDI) model,^28^ aim to characterize the complexity of cerebral tissue by decomposing the dMRI signal into compartments with distinct diffusion environments. NODDI consists of an intraneurite compartment, capturing the diffusion inside dendrites and axons, an extraneurite compartment, representing diffusion in and around glial cells and neuronal somas, and CSF (Fig. 1b). The density of dendrites and axons is quantified with the neurite density index (NDI). The dispersion of fiber populations in the intraneurite compartment is quantified with the orientation dispersion index (ODI). ODI is low in highly parallel microstructural environments such as coherent white matter fibers in the posterior limb of the internal capsule or the corpus callosum, and high in regions with crossing fibers like the centrum semiovale.

To date, the use of NODDI and fixel-based metrics in studies characterizing the relationship between everyday exposures and early brain development are limited. However, these promising techniques will help refine our understanding of microstructural changes associated with prenatal exposures.

dMRI analysis can be undertaken on a voxel-wise basis using methods such as tract-based spatial statistics^29^ which characterizes associations within the center of white matter tracts, or with region-of-interest (ROI) analyses in which dMRI metrics are extracted from a-priori hypothesized regions. While some studies use a mask derived from structural MRI segmentations to define ROIs (e.g., the amygdala), dMRI tractography enables the non-invasive reconstruction of white matter tracts of interest based on the underlying dMRI signal (Fig. 1b right). DTI, NODDI and fixel-wise metrics can be extracted from reconstructed white matter tracts for further analysis. Finally, while dMRI is typically used to investigate white matter microstructure, DTI and NODDI metrics have previously been used to characterize the maturation of cortical microstructure in infants.^30^ However, to our knowledge no study has assessed the relationship between cortical microstructure and everyday exposures.

### Functional MRI

Functional MRI can improve our understanding of the impact of antenatal stressors on the development of brain network function. Functional MRI provides indirect information about brain activity through the blood oxygenation level dependent (BOLD) contrast. As hemoglobin has different magnetic properties when bound to oxygen, it acts as an endogenous contrast agent that can be measured with a T2* weighted sequence.^31^ By repeatedly sampling the BOLD signal, relative increases and decreases of the signal can be interpreted as local changes in neural activity.^32^ In fetuses and neonates, functional brain development is typically assessed with resting state functional MRI (rs-fMRI), in which fluctuations of the BOLD signal at rest are measured. Low-frequency fluctuations occur in a highly correlated fashion across spatially distinct brain regions and are thought to represent intrinsic functional brain networks.^33^

Seed-based functional connectivity was the first method used to characterize rs-fMRI brain networks^33^ and is the most commonly used rs-fMRI analysis in the everyday exposure literature (Fig. 1c). The average signal fluctuations over the acquisition period (termed a time series, see Fig. 1c for an example) within an ROI, is correlated with time series from all other voxels across the brain or average time series within other ROIs. The degree of correlation between the seed and other voxels/ROIs is taken as the strength of functional connectivity. However, seed-based functional connectivity measures are highly dependent on ROI placement and size.^34^

Data-driven techniques have been developed to assess the relationship between rs-fMRI network development and everyday exposures. Equally sized ROIs across the brain can be defined using data-driven methods.^35^ The BOLD signal from all voxels in the brain can also be spatially and temporally decomposed into networks using data-driven techniques such as multivariate exploratory linear optimized decomposition into independent components (MELODIC)^36^ (Fig. 1c), which uses independent component analysis. Another technique is Leading Eigenvector Dynamics Analysis^37^ (LEiDA), which characterizes dynamic functional connectivity by assessing dominant connectivity patterns across the brain over time, taken as the leading eigenvector of BOLD phase coherence patterns in each ROI over each repetition time of the acquisition. This method captures transient connectivity patterns and changes in network behavior over time.

Finally, both structural (Fig. 1b) and functional (Fig. 1c) networks across the brain can be reconstructed using a parcellation to define ROIs (termed ‘nodes’) and rs-fMRI time series correlations,^38^ dMRI microstructure metrics^39^ or weighted streamline volume estimates^40^ to define the degree of connectivity between regions (‘edges’). Connectomics encompasses a set of analyses designed to interrogate the global and local organizational properties of these brain networks.^41,42^ The relationship between these organizational properties and early life exposures can be assessed.

## Everyday exposures

We discuss key MRI studies reporting differences in fetal and neonatal brain development associated with prenatal everyday exposures. Our focus is on parental psychosocial stress, which encompasses negative psychological wellbeing including depression, anxiety, and stressful experiences during pregnancy and pre-conception, as well as on environmental toxicants such as pollution and lead exposure, and socioeconomic adversity. The terms used throughout this review reflect the terminology used by the authors of the included studies.

### Maternal stress during pregnancy

An increasing number of neuroimaging studies are aiming to investigate the neural correlates of in-utero exposure to maternal stress,^9,43^ Most research in this field has been done using data from large multimodal cohort studies such as the dHCP,^44^ Growing up in Singapore Towards Healthy Outcomes (GUSTO),^45^ Baby Connectome Project,^46^ and FinnBrain.^47^

While some studies have focused on diagnosed mental health conditions, most report differences associated with subclinical levels of depression, anxiety, and stress, highlighting that prenatal stress can be considered an ‘everyday exposure’. Studies of prenatal maternal depression are typically based on data from self-report questionnaires such as the Edinburgh Postnatal Depression Scale (EPDS),^48^ the Center for Epidemiologic Studies Depression Scale (CES-D)^49^ or the Hamilton Rating Scales for Depression (HAM-D).^50^ Studies of maternal anxiety and stress are typically based on self-report measures such as the State Trait Anxiety Inventory (STAI)^51^ and the Perceived Stress Scale (PSS).^52^ Given the high co-occurrence of these conditions/symptoms^53^ and the limited number of studies accounting for multiple exposures, it is difficult to establish whether and how their impact on the developing brain may differ.

#### Structural MRI

Most prenatal stress imaging studies are based on segmentations, with many focusing on limbic system ROIs. In a South African birth cohort (*n* = 124), infants exposed to maternal prenatal depression had larger volumes in the right amygdala and bilateral caudate nucleus, while hippocampal volume was larger in female infants only.^54^ Sex-specific associations were also reported in a neonatal sample from the FinnBrain cohort, where pregnancy-specific anxiety was positively associated with left amygdala volume in girls only^55^ (*n* = 122). The same research group used a composite measure of maternal psychological distress measured at 3 time-points during pregnancy (14, 24 and 34 weeks gestational age (GA)) and reported that newborn sex moderated the relationship between 24 weeks psychological distress and left and right amygdala volumes (decreased in males).^56^ There were no associations with hippocampal volume in either of the two FinnBrain studies, in line with findings from a study of preterm infants (*n* = 221) reporting no associations between maternal trait anxiety/stressful life events and volumes in hippocampus (as well as frontal and temporal lobes, amygdala, and thalamus).^57^ These null results were based on segmentations of bilateral regions-of-interest and were supported by an exploratory analysis using tensor-based morphometry. Bezanson and colleagues^58^ also reported no associations between maternal anxiety and depression and regions in the limbic system such as the hippocampus and amygdala. However, in this sample, maternal state anxiety was positively associated with infant right pallidum volumes, but only when maternal depression was also included in the model.

Longitudinal neuroimaging could shed light on potential reasons for some of the inconsistencies and null results reported in the neonatal structural MRI literature. In a ROI study from the GUSTO cohort (*n* = 175), there was no relationship between maternal anxiety at 26 weeks GA and bilateral hippocampal volumes.^59^ However, in a subsample scanned at 6 months of age (*n* = 35), hippocampal volume was smaller in infants exposed to prenatal anxiety, controlling for postnatal anxiety. Further, another study from the GUSTO cohort (*n* = 167) reported a positive association between left amygdala volume and cortical thickness in a region of the posterior insula at birth, in females (but not males) whose mothers experienced prenatal depressive symptoms.^60^ At 4.5 years there was a negative association between right amygdala volume and thickness in a region of the left inferior frontal gyrus in females whose mothers experienced prenatal depressive symptoms.

While MRI in infancy minimizes postnatal influences, it does not completely eliminate them, highlighting the necessity of MRI studies focusing on the fetal brain. Wu and colleagues^61^ (*n* = 119) reported that anxiety and perceived stress were associated with fetal brain gyrification in the temporal and frontal lobes, while trait anxiety specifically was associated with lower left hippocampal volume. A longitudinal study from the same authors^62^ (*n* = 97) reported associations between prenatal psychological distress, fetal left hippocampal volume, cortical gyrification and sulcal depth, and outcomes in infancy. Further, the same research group^63^ compared fetuses who were in utero during the COVID-19 pandemic (*n* = 65; without known COVID-19 exposures), with pre-pandemic fetuses (*n* = 137). The pandemic cohort exhibited lower volumes in fetal white matter, hippocampus, and cerebellum, decreased cortical surface area and gyrification in all four lobes, as well as decreased sulcal depth in frontal, parietal, and occipital lobes. Anxiety and depression were higher in the pandemic group, compared to the pre-pandemic group. Key findings include negative associations between stress/anxiety and hippocampal and cerebellar volumes, as well as positive associations between depression/anxiety and frontal lobe sulcal depth. Lastly, after controlling for multiple comparisons, De Asis-Cruz and colleagues^64^ reported no significant associations between maternal psychological distress and fetal cortical thickness (*n* = 177).

#### Diffusion MRI

Most dMRI studies in the field have used DTI and focused on limbic system ROIs (e.g., amygdala microstructure, limbic-prefrontal white matter tracts). In the FinnBrain cohort, depressive symptoms were associated with higher MD in the left amygdala sex-dependent way (i.e., in boys).^65^ Amygdala microstructure was also the focus of an earlier GUSTO study (*n* = 157) reporting that higher depressive symptoms were associated with differences in neonatal right amygdala microstructure (i.e., lower FA and AD) but not volume.^66^ In a smaller sample (*n* = 54), the same authors later reported relationships between anxiety scores and FA in multiple brain areas (including right insula, dorsolateral prefrontal cortex, right middle occipital cortex, right angular gyrus, uncinate fasciculus, posterior cingulate, and parahippocampus).^67^ In another small neonatal sample (*n* = 34) both maternal anxiety and depression negatively correlated with FA values in left and right prefrontal white matter, left and right middle frontal gyrus white matter, and fornix.^68^ Further, Demers and colleagues^69^ reported that higher state anxiety at 29 weeks GA was associated with increased FA and AD in the right anterior cingulate. Lastly, a US study using DTI and NODDI reported associations between a composite score of anxiety and depression and altered microstructure (i.e., decreased ND and increased MD, RD, and AD), particularly in right frontal white matter, with results varying by infant sex (lower FA and ND in females, and higher FA and ND in males across several regions).^70^

**Fig. 2:**
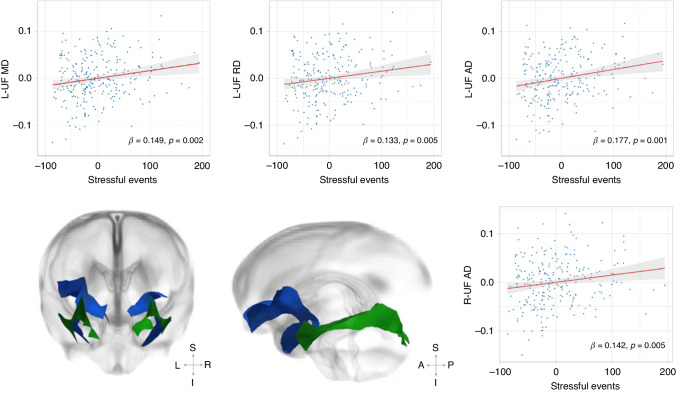
The relationship between maternal depressive symptoms and fibre diffusivity. Plots showing the relationship between maternal depressive symptoms (EPDS) and mean fiber diffusivity (FD) in the left and right uncinate fasciculus, controlling for infant gestational age at birth, postmenstrual age at scan, sex, maternal socioeconomic status, and maternal history of poor mental health. Adapted from Lautarescu and colleagues^71p^ublished under Creative Commons Attribution Licence CC BY 4.0 (https://creativecommons.org/licenses/by/4.0/).

To our knowledge, only one study in this field has used fixel-based fiber metrics of multishell HARDI. In a sample of 413 mother-infant dyads enrolled in the dHCP, higher maternal depressive symptoms were associated with increased infant FD in the uncinate fasciculus bilaterally. There were no associations with cingulum microstructure. Depressive symptoms and higher left uncinate fasciculus FD were associated with more socio-emotional difficulties in toddlerhood^71^ (Fig. 2). Results were supported by an exploratory analysis using DTI, suggesting the same direction of effect (i.e., increased FA and decreased MD). As the direction of effect suggests white matter maturation, the results may indicate an accelerated developmental trajectory in infants exposed to maternal depression.

Structural brain connectivity was assessed in one study,^72^ in which prenatal depressive symptoms were negatively associated with changes in the default mode network organization assessed with graph theory metrics. Infant sleep moderated the association between prenatal depression and network organizational properties.

**Fig. 3:**
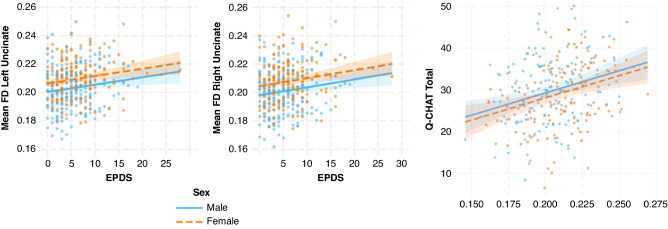
The relationship between stressful life events and diffusion MRI measures. Partial regression scatterplots showing the relationships between stressful life events and mean diffusivity (MD), axial diffusivity (AD), and radial diffusivity (RD) in the left uncinate fasciculus (L-UF; top row) and AD in the right uncinate fasciculus (R-UF; bottom row). These results were obtained controlling for infant gestational age at birth, postmenstrual age at scan, socioeconomic status, total days on parenteral nutrition, sex, and maternal age. Bottom left: Glass brain” illustrations showing the skeletonized versions of the uncinate fasciculus (blue) and inferior longitudinal fasciculus (green) medial surface overlaid on the template radial diffusivity image, presented in coronal and sagittal planes (left to right). Adapted from Lautarescu and colleagues^73^ published under Creative Commons Attribution Licence CC BY 4.0 (https://creativecommons.org/licenses/by/4.0/).

Literature examining the impact of prenatal everyday stressors on babies who are already at risk for poor neurodevelopmental outcomes (e.g., due to pregnancy complications or congenital abnormalities) is limited. In a large sample of preterm infants scanned at term equivalent age (*n* = 251), maternal stressful life events in the year prior to birth (but not trait anxiety) were associated with higher AD, RD and MD in the left uncinate fasciculus, and higher AD in the right uncinate fasciculus, determined using diffusion MR tractography (Fig. 3).^73^ Further, in a structural MRI study using vertex-wise deformation analysis, maternal prenatal stress and anxiety were associated with smaller volumes in the left and right hippocampus and cerebellum in mothers whose fetuses had congenital heart disease (CHD), but not in mothers carrying healthy fetuses.^74^

#### Functional MRI

Functional MRI studies in this field are largely based on seed-to-voxel or seed-to-seed connectivity, primarily using limbic system ROIs. Neonatal amygdala functional connectivity was investigated in a recent study assessing maternal stress, anxiety, and depression at several prenatal timepoints.^75^ Infants of mothers who experienced increasing levels of stress later into the pregnancy exhibited stronger functional connectivity between the amygdala and the anterior insula and ventromedial prefrontal cortex. These results are in line with those of Na and colleagues^76^ who also reported associations between stressors later in pregnancy (i.e., depression in the third trimester only) and infant functional connectivity in the frontal lobe and between the frontal and temporal lobes and the occipital lobe.

**Fig. 4:**
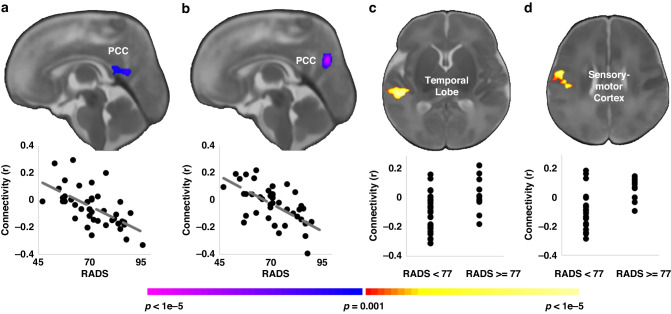
Association of maternal depression and hippocampal connectivity. Higher depressive symptoms in the 3rd trimester as measured by the Reynolds Adolescent Depression Scale (RADS) were associated with weaker infant connectivity between the **a** left and **b** right hippocampus and the posterior cingulate cortex (PCC). When dichotomized based on clinical cutoffs, neonates from mothers with prenatal depression exhibited greater connectivity **c** between the left hippocampus and the right temporal lobe and **d** between the right hippocampus and the right sensory-motor cortex. Scatterplots below the images visualize the distribution of the observed data points for average infant connectivity in the detected regions plotted against maternal depressive symptoms or clinical group. Image from Scheinost and colleagues^79^ published under Creative Commons Attribution Licence CC BY 4.0 (https://creativecommons.org/licenses/by/4.0/).

In a small sample, preterm infants had reduced connectivity between the amygdala and several regions, compared to infants born at term, suggesting that this structure may already be vulnerable to the impact of prenatal stressors^77^ Further, preterm infants exposed to prenatal stress showed reduced functional connectivity between the amygdala and the thalamus, hypothalamus, and peristriate cortex. A study conducted by the same author group^78^ investigated hippocampal functional connectivity and multiple types of distress in a small sample of pregnant adolescents (*n* = 42). Maternal 3rd trimester distress was associated with weaker connectivity with the cingulate cortex, and stronger connectivity with the temporal lobe (Fig. 4). However, each dimension of maternal distress was associated with a unique region within the cingulate or temporal lobe. Lastly, a recent analysis using LEiDA for the first time in infants (*n* = 20) reported that prenatal distress (composite score of depression and anxiety) was associated with the stability of a frontoparietal network.^79^

Evidence from fetal rs-fMRI is limited. In a longitudinal study, higher maternal stress (combining multiple measures) was associated with lower fetal cerebellar-insular functional connectivity and disturbed sleep in toddlerhood.^80^ In another study of 50 fetuses, anxiety was associated with altered functional connectivity across several brain regions.^81^

#### Multimodal MRI

Several studies have reported findings based on multimodal MRI data. In a small sample of infants (*n* = 33), prenatal stress was associated with decreased functional connectivity and increased structural connectivity between the amygdala and medial prefrontal cortex (PFC), even after controlling for preconception stress.^82^ Neonatal rs-fMRI and dMRI were also combined by Posner and colleagues,^83^ with maternal depression associated with decreased structural connectivity between right amygdala and right ventral PFC and negative functional connectivity between right amygdala and dorsal prefrontal cortex bilaterally. As part of the Pregnancy during COVID-19 Pandemic cohort study, Manning and colleagues^84^ reported associations between maternal prenatal distress and amygdala-prefrontal connectivity measures (rs-fMRI and dMRI), with social support as a significant moderator. Pandemic related stress was also examined in a US fetal cohort,^85^ where increased perceived stress was associated with higher brainstem volume and reduced temporal variance of the BOLD signal from the whole brain.

#### Biological and Genetics findings

Several neonatal MRI studies have incorporated genetics data into prenatal stress studies. Evidence from GUSTO suggests neonatal genomics moderate the relationship between prenatal depression and neonatal brain development. Genetic variants in a gene involved in regulating the Hypothalamic–Pituitary–Adrenal axis (i.e., FKBP5) moderated the association between maternal depressive symptoms and right hippocampal volume.^86^ In an overlapping sample, neonatal BDNF genotype influenced the association between maternal anxiety and epigenome at birth, and between the epigenome and neonatal brain structure.^87^ Another GUSTO study reported a moderating effect of neonatal genomic profile risk score for major depressive disorder on the relationship between maternal prenatal depressive symptoms and right amygdala volume.^88^ While the moderating effect was significant across the US and Singapore cohorts included in this study, the direction of effect differed, potentially due to different patterns of genomics and other exposures between the cohorts. Two FinnBrain studies investigating maternal depression^89^ and anxiety^90^ reported genotype-by-environment effects on infant volumes in the limbic system, with exploratory analyses suggesting sex-specific effects.

Several studies used biological indicators of prenatal stress, such as cortisol and inflammation. Elevated maternal cortisol levels are associated with amygdala microstructure, structural connectivity^91^ and functional connectivity,^92^ as well as hippocampal connectivity.^78^ Maternal inflammation as measured by interleukin-6 levels (IL-6) is associated with amygdala volume and connectivity,^93^ microstructural differences in the uncinate fasciculus,^94^ and differences in functional connectivity.^95,96^

### Paternal stress and preconception stress

While prenatal stress has typically been conceptualized as in utero exposure to maternal stress, an increasing number of studies have started to focus on preconception stress, of not only mothers but also fathers.

Even when controlling for maternal distress during pregnancy, maternal childhood adversity has been associated with differences in infant brain structure, such as lower neonatal intracranial volume^97^ and connectivity such as infant fronto-amygdala connectivity^98^ and fetal amygdala functional connectivity.^99^ Different types of adversity have been proposed to impact the developing brain in different ways. In a study by Lyons-Ruth and colleagues,^100^ lower infant gray matter volume was reported in association with maternal history of neglect (but not abuse), while a history of abuse (but not neglect) was associated with decreased volume in the amygdala in older infants only. The impact of maternal early life stress on neonatal brain may also differ based on infant sex, with Lugo-Candelas and colleagues^101^ reporting increased intra-hemispheric frontal-limbic connectivity for males whose mothers experienced childhood maltreatment, but not females. Frontal-limbic connectivity was later associated with childhood somatic complaints in males only.

Paternal stress can impact offspring via changes to gametes,^102^ but research on the impact of paternal stress on neonatal and fetal early brain development is limited. In a small FinnBrain study (*n* = 72), paternal early life stress was associated with increased FA values in the body of the corpus callosum, right superior corona radiata, and parts of the internal capsule,^103^ even when controlling for confounders including maternal early life stress, maternal SES, or prenatal depression. A recent preprint^104^ reported positive associations between maternal (but not paternal) childhood maltreatment and infant left amygdala volume. In an exploratory analysis, the timing of exposure was considered, with increased left amygdala volume observed in infants whose mothers experienced childhood maltreatment between ages 13 and 18, and in those whose fathers experienced this between ages 0 and 6.

### Environmental toxicants

While the majority of literature examining everyday exposures and fetal and neonatal brain development has assessed the impact of prenatal stress, there are a small number of studies examining the influence of exposure to environmental toxicants. Epidemiological and large cohort studies have highlighted the effect of in utero exposure to neurotoxicants including heavy metals^105–107^ and air pollution^108–110^ on childhood neurodevelopmental outcomes. In animal studies, prenatal exposure to pollution is associated with altered microglial development, disrupted cortical development, and altered myelination.^111,112^ Animal studies suggest early life exposure to heavy metals such as lead impairs neuronal development, hippocampal neurogenesis and astrocytic function.^113–115^

Brain MRI provides novel opportunities to elucidate the mechanisms through which in utero exposure to environmental toxicants impacts neurodevelopmental outcomes. A recent systematic review reports that in utero exposure to environmental toxicants, including air pollution and heavy metals, is associated with altered microstructural, morphological and functional brain development in childhood.^116^ However, research examining the relationship between prenatal exposure to air pollution or heavy metals and fetal or neonatal brain development is limited.

**Fig. 5:**
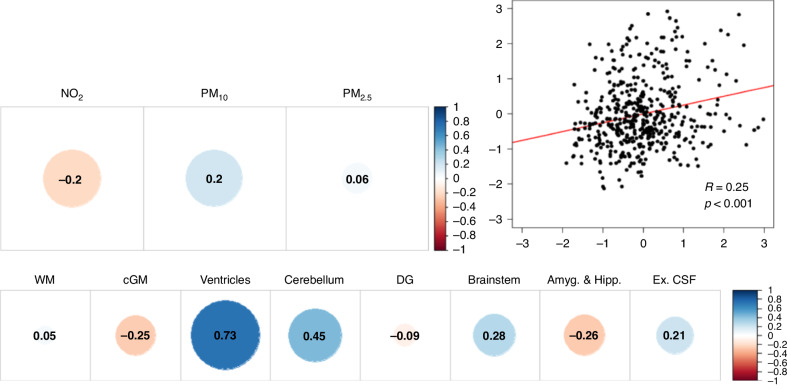
Canonical correlations between air pollutants and relative brain volumes. Canonical correlations of air pollutants: NO_2_, 2. PM_10_, 3. PM_2.5_, canonical correlations of the relative brain volumes: White Matter, Cortical Gray matter, Ventricles, Cerebellum, Deep Gray Nuclei, Brainstem, Amygdala & Hippocampus, extracerebral CSF. A Scatterplot of the canonical correlation. Adapted from Bos and colleagues,^118^ published under Creative Commons Attribution Licence CC BY 4.0 (https://creativecommons.org/licenses/by/4.0/).

To our knowledge, the only study to date to assess the relationship between prenatal exposure to air pollution and neonatal brain morphology investigated segmented brain volumes from 469 healthy infants born at term between the years 2015 to 2020 and recruited to the developing human connectome project (http://www.developingconnectome.org/).^117^ Canonical correlation analysis showed that higher gestational exposure to particulate matter with a diameter of 10 microns or less (PM_10_) and lower exposure to NO_2_ was associated with larger relative ventricular and cerebellar volume, along with modest associations with smaller relative cortical gray matter, amygdala and hippocampus volumes, and larger relative volumes of brainstem and extracerebral CSF (Fig. 5).

A 2024 study of 101 mother-infant dyads reported higher prenatal urinary lead levels were associated with smaller bilateral neonatal amygdala volumes. Interestingly, the relationship between prenatal lead levels and left amygdala volume was unique to participants with low depressive symptoms or high family income.^118^

A small fetal whole-brain rs-fMRI study assessing functional connectivity across data-driven ROIs across the brain reported that exposure to lead during pregnancy (lead naïve *n* = 13, lead exposed *n* = 13) was associated with weaker interhemispheric functional connectivity and stronger right hemispheric anterior-posterior connectivity with advancing GA.^119^

Further studies are required to further our understanding of the impact of air pollution and lead exposure and brain development in the fetal and neonatal period. To our knowledge the impact of prenatal exposure to other environmental toxicants such as manganese and phthalates on the fetal or neonatal brain has not been investigated, despite evidence linking these toxicants to altered brain development in children.^116^ Future research with large samples that include other MR methods and toxicant exposures are required. In addition, longitudinal studies including neurodevelopmental outcomes are necessary to fully characterize the relationship between in utero exposure to environmental toxicants, early brain development, and later neurodevelopmental outcomes.

### Socioeconomic status

High rates of prenatal stress and environmental toxicants are often present in conjunction with other social determinants of health such as low socioeconomic status (SES), which is in turn often associated with other adverse circumstances including reduced prenatal care, poor nutrition, financial difficulties, insecure housing, and increased prevalence of tobacco, alcohol, and substance use.^120^ These stressors have the potential of directly affecting brain development but may also cause or exacerbate parental stress. There is a large body of work highlighting the relationship between SES and childhood outcome, particularly cognitive performance but also psychomotor function and psychopathology.^121–127^

To date, few studies have assessed the relationship between brain structure and SES in the newborn period. In a small neonatal sample (*n* = 37), low maternal SES was associated with increased local volumes at the surface of the right occipital lobe, left temporal pole, left inferior frontal and anterior cingulate regions.^128^ This association was moderated by social support (marital/partner status). In this longitudinal study, SES was also associated with language and behavioral outcomes at 24 months. Lower SES has also been associated with volume differences in the amygdala,^129^ hippocampus,^129^ cortical gray matter^130–132^ and deep gray matter,^132^ as well as white matter volumes and cortical folding.^132^ Qiu and colleagues^88^ reported that the relationship between socioeconomic status and right amygdala and hippocampal volume and shape was moderated by infant genomic profile risk score for depression. The interaction effect on the right hippocampus remained significant after controlling for maternal depression. There is a need for further studies that model explicitly the interaction between SES and other environmental and familial factors on fetal and newborn brain development.

In another study using dMRI, social disadvantage, but not psychosocial stress, was independently associated with lower MD in the bilateral inferior cingulum bundle and left uncinate, right fornix, and lower MD and higher FA in the right dorsal cingulum bundle.^133^ Low SES is also associated with differences in infant brain function. In a study of term and preterm born infants^134^ (*n* = 112), lower SES was associated with changes in functional connectivity (primarily in striatum and medial PFC), with connectivity changes mediating the relationship between SES and behavioral outcomes in toddlers.

While SES is often examined with variables such as income and education, it is important to also consider other co-occurring factors. For example, a recent multimodal study of maternal exposure intimate partner violence in a South African cohort^135^ reported sexually dimorphic effects in diffusivity measures, as well as volumetrics, with larger caudate nucleus volumes in males only, and smaller amygdala volumes in females only. Further, living in a high-crime neighborhood was associated with weaker neonatal limbic and frontal functional brain connections,^136^ while experiences of discrimination and acculturation have been associated with amygdala functional connectivity.^137^

## Conclusions and future directions

This narrative review discusses how fetal and neonatal neuroimaging has improved our understanding of the impact of prenatal stress and environmental toxicants on early brain development. A large body of literature has assessed associations between neonatal brain development and (sub)clinical levels of depression, anxiety or stressful experiences across large cohorts. The most widely used techniques in this body of literature are region of interest volumetric and DTI studies. Prenatal stress has been associated with differences in volumes in the hippocampus, amygdala and basal ganglia nuclei, and altered microstructure of the amygdala, cingulum bundle, fornix, uncinate fasciculus and frontal white matter, often in a sexually dimorphic manner. This is complemented with rs-fMRI literature reporting altered amygdala and frontotemporal connectivity in response to maternal stress. However, the evidence is mixed, with some studies reporting opposing relationships or null results. Research using fetal MRI is sparse, but recent advances in this area will facilitate a more complete understanding of how stress exposure impacts brain development. Many of the reported changes associated with prenatal stress are within limbic structures, however, it is important to note that many of these results are from studies selecting limbic system ROIs, and studies incorporating whole brain analyses have reported macrostructural, microstructural and functional changes outside the limbic system. Large studies incorporating measures of prenatal stress and brain development at multiple fetal, neonatal and infant time points may disentangle these mixed effects and elucidate critical periods in which stress exposures alter volumetric and microstructural brain development in different infants. In addition, newer dMRI methods including fixel-based and NODDI metrics as well as structural connectivity analyses have started to refine our understanding of how microstructural brain development is altered by stress exposure. A few studies have reported associations between cortical morphometry alterations and stress exposure, particularly in the fetus. However, the literature is sparse, and further research is needed to fully characterize the influence of stress exposure on cortical development across the fetal and neonatal periods.

New literature has highlighted the potential effects of prenatal air pollution and lead exposure on fetal and neonatal brain development, which may represent important avenues for public health interventions. However, the literature is sparse and further research is needed to fully characterize the influence of environmental toxicants on brain development in the fetal and neonatal period. A growing body of literature is widening our understanding of the relationship between early brain development and everyday exposures by incorporating biological measures of parental stress, (epi) genomics, paternal exposures, preconception stressors and environmental toxicants such as air pollution. By combining these measures with advanced brain MRI analysis, the potential exists to revolutionize our understanding of how the parental and offspring exposome affects longitudinal brain development.

Large cohort studies that use multimodal MRI at multiple timepoints as well as longitudinal follow-up have improved our understanding of the relationship between everyday exposures, altered brain development, and later outcomes. Importantly, many of these datasets are open access, providing pathways for further advancements by combining datasets and using novel analysis techniques. This will facilitate comprehensive longitudinal research studies which characterize the cascading effects of prenatal exposures on early brain development and subsequent effects on childhood outcomes. Further, in order to enable researchers to draw useful conclusions from the neuroimaging literature within the field of everyday exposures, there is an urgent need for transparent reporting of all research findings (including null results), as well as systematic reviews and meta-analyses.

Psychological stressors frequently co-occur with socioeconomic deprivation and exposure to environmental toxicants, but few studies investigate the impact of multiple stressors on early brain development. Our narrative review encourages researchers to consider stress from a holistic point of view, investigating the overlapping and potentially additive effects of maternal and paternal mental health, socioeconomic deprivation, and environmental toxicants on early brain development. In addition, the impact of everyday exposures on early brain development can be influenced by many other factors (e.g., timing and duration of exposure, infant sex, pre-existing vulnerabilities, factors associated with resilience such as high socioeconomic status). Future neuroimaging studies that include comprehensive assessments of mental health, other stressors such as environmental toxicants, and potential mediators and moderators are required to understand the impact of multiple stressors and may provide key information to enable the development of future interventions and support strategies.

An important emerging avenue of research is the impact of prenatal stress on the brain in fetuses and neonates who are vulnerable to altered brain development, such as those born prematurely or with CHD. However, to our knowledge, the impact of prenatal environmental toxicant exposure on early brain development in at-risk groups has not been investigated.

The literature regarding prenatal stress often reports increases, decreases and non-significant changes in MR metrics with increased exposure. When assessing the relationship between a stressor and brain development, it is assumed that the effect of the exposure is uniform across individuals. However, the impact of stressors on brain development in individual mother-infant dyads may be influenced by differences in risk and resilience factors, physiology, and (epi)genomics. Normative modeling approaches aim to understand differences at the level of an individual while mapping these differences in relation to a reference population in a process akin to using a pediatric growth chart.^138^ Normative modeling has revealed heterogeneous patterns of altered brain development in infants born prematurely,^139–141^ and those with Down syndrome^142^ and CHD.^143^ These techniques provide novel opportunities to move beyond group effects and characterize the relationship between everyday exposures and brain development in individuals.

In the future, using deep learning methods on large cohorts that incorporate multiple everyday exposures and brain measures may enable comprehensive investigations into the complex relationships between everyday exposures, early brain development and later outcomes, which are not feasible with traditional statistical techniques.

Although the focus of this review is neuroimaging, there is clear evidence of a shared brain-placental axis during pregnancy.^144^ There is evidence that everyday exposures alter the placental epigenome, with consequences for infant outcomes such as birth weight.^145–147^ Saeed and colleagues^148^ reported that differences in quantitative MR measures of placental structure in pregnant women during the COVID-19 pandemic and pre-pandemic controls were mediated by the level of prenatal maternal stress exposure. Future research combining MR of the brain and placenta, together with assessment of multiple prenatal exposures and other modalities such as placental epigenomics could enhance our knowledge of the mechanisms underlying the relationship between prenatal exposures and offspring brain development.

Finally, advances in MR hardware, moving beyond conventional field strengths (1.5 or 3 Tesla(T)) have opened new avenues for fetal and neonatal brain development. Conventional MR scanners are typically situated in hospitals in high resource settings due to infrastructure requirements such as access to liquid helium and reliable high power electricity. New low (0.55 T) and ultra-low (0.064 T) field systems require less infrastructure and can be used to acquire fetal^149^ and neonatal^150^ brain MRI. Large international studies are currently underway to investigate how ultra-low field brain MRI can be utilized in low resource settings (e.g., https://www.unity-mri.com/). In the future, this technology will enable research into the relationship between everyday exposures and early brain development in wider populations, including low- and middle-income countries. In the future, this technology will enable research into the relationship between everyday exposures and early brain development in low resource settings. Advances in ultra-high field (7 T) MRI, mean that neonatal neuroimaging^151^ can be undertaken at sub-millimeter resolutions, which will provide insights into layer and sub-nuclei specific changes in functional and structural development related to everyday exposures.
